# Supplementary material for: GABA, glutamate dynamics and BOLD observed during cognitive processing in psychosis patients with hallucinatory traits
Source: Sci Rep. 2025 Jun 3;15:19466. doi: 10.1038/s41598-025-03644-x (PMC12134229; doi:10.1038/s41598-025-03644-x)
Supplement: Supplementary file 1 — Supplementary Material 1 [file 41598_2025_3644_MOESM1_ESM.pdf]

A Additional Subject Details

	n	ICD-10	Diagnosis
**Schizophrenia**	**29**		
	23	F20.0	Paranoid schizophrenia
	3	F20.3	Undifferentiated schizophrenia
	1	F20.4	Post-schizophrenic depression
	2	F20.9	Schizophrenia, unspecified
**Other Schiz. Spectrum**	**7**		
	1	F23.3	Acute paranoid psychosis
	2	F25.0	Schizoaffective disorder, manic type
	1	F25.1	Schizoaffective disorder, depressive type
	3	F29	Unspecified non-organic psychosis
**Mood/affective**	**2**		
	1	F31	Bipolar disorder
	1	F32.3	Severe depressive episode with psychotic symptoms
**Personality/behavioural**	**4**		
	1	F60.0	Paranoid personality disorder
	1	F60.3	Emotionally unstable personality disorder
	1	F61.0	Mixed and other personality disorders
	1	F62.8	Other enduring personality changes
**Drug-induced psychosis**	**4**		Mental and behavioural disorders due to…
	1	F12.5	Use of cannabinoids; psychotic disorder
	2	F19.0	Multiple drug use and use of other psychoactive substances; acute intoxication
	1	F19.52	…psychosis, mainly with hallucinations
**Other**	**5**		
	1	F06.0	Organic hallucinosis
	1	F90	Disturbance of activity and attention
	2	N/A	Unknown diagnosis
	1	N/A	No diagnosis

Supplementary Table 1 Diagnoses of patients in the present study, according to ICD-10 criteria

Diagnoses are according to the ICD-10 criteria, described in the following publications (English and Norwegian translation):

World Health Organization. (1992). *The ICD-10 classification of mental and behavioural disorders: Clinical descriptions and diagnostic guidelines* (Reprinted). World Health Organization. https://www.who.int/docs/default-source/classification/other-classifications/bluebook.pdf

World Health Organization. (2016). *ICD-10 psykiske lidelser og atferdsforstyrrelser: Kliniske beskrivelser og diagnostiske retningslinjer* (10. rev., 19. oppl). Universitetsforlaget. https://www.ehelse.no/kodeverk-og-terminologi/ICD-10-og-ICD-11

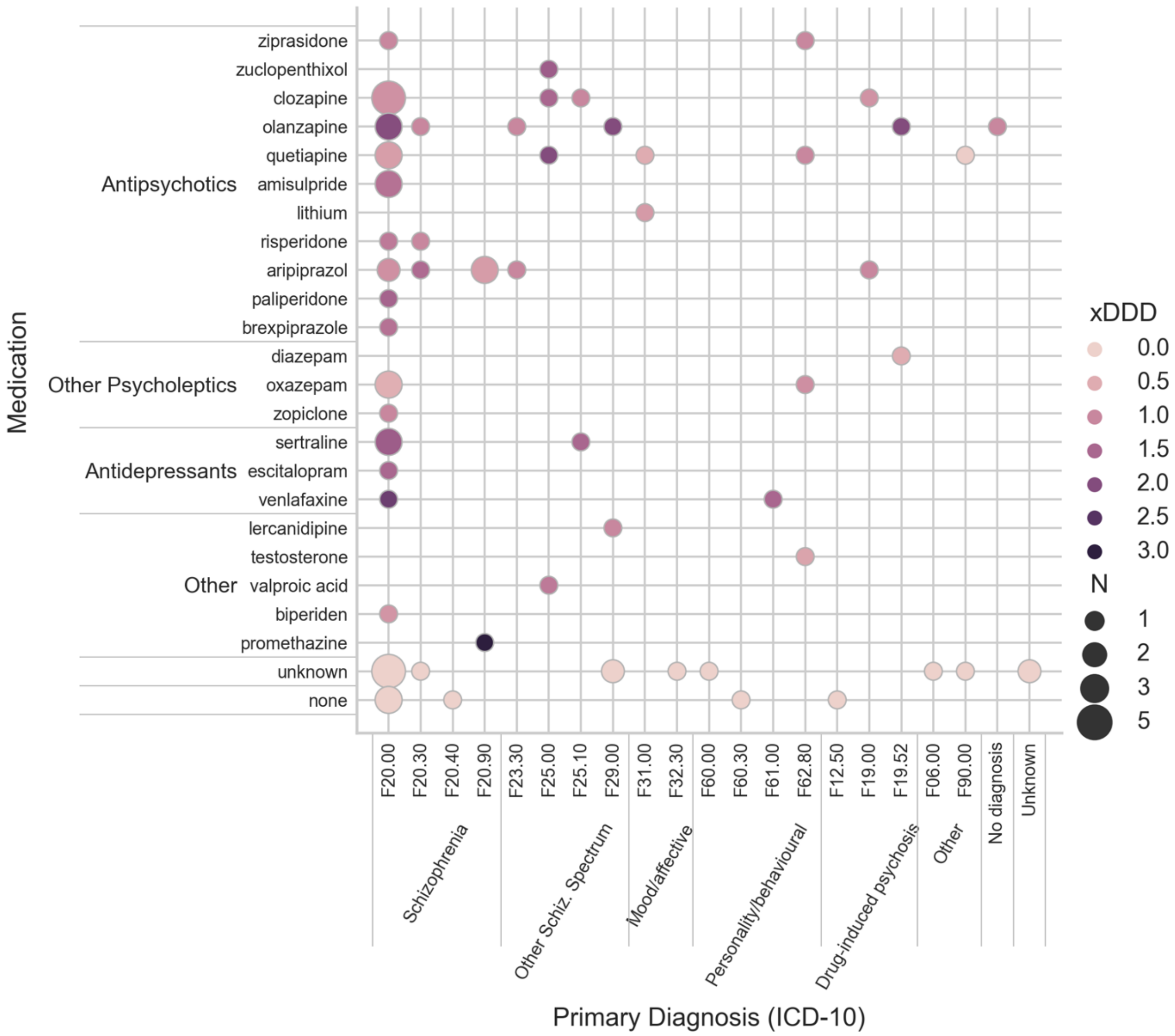

Supplementary Figure 1 Medication and mean of the (maximum) prescribed dosage, expressed relative to the defined daily dose (DDD). Size of the points is proportional to the number of patients (N), with darker shading indicating higher dosage (xDDD).

In several cases, the prescribed dosage was qualified with terms such as "up to" and "as needed" ("opp til", "ved behov"); some subjects reported that they had not taken prescribed medicine in the period leading up to the study. Dosage presented here therefore represents the maximum prescribed dosage, and is likely to slightly overestimate the actual dosage taken.

Defined daily dose (DDD) according to WHO Collaborating Centre for Drug Statistics Methodology, ATC classification index with DDDs, 2024. Oslo, Norway 2024, searchable online at https://atcddd.fhi.no/atc_ddd_index/

ATC codes were derived from trade names via the register at https://www.felleskatalogen.no/medisin/atc-register/

B MRSinMRS checklist

Site (name or number)	Haukeland University Hospital
**1. Hardware**	
a. Field strength [T]	3 T
b. Manufacturer	GE HealthCare
c. Model (software version if available)	MR 750, DV 28
d. RF coils: nuclei (transmit/receive), number of channels, type, body part	GEHC 8-channel ^{1}H head coil
e. Additional hardware	NNL visual system, response grips and SyncBox for functional task
**2. Acquisition**	
a. Pulse sequence	MEGA-PRESS (GABA+ editing, ATSM patch), adapted for triggering and with additional water-unsuppressed reference scans (every third FID)
b. Volume of interest (VOI) locations	Anterior Cingulate Cortex
c. Nominal VOI size [cm3,mm3]	22 x 36 x 23 mm^3 (18.2 mL)
d. Repetition time (T_R), echo time (T_E) [ms, s]	T_R = 1500 ms, T_E = 68 ms
e. Total number of excitations or acquisitions per spectrum<br>In time series for kinetic studies<br>i. Number of averaged spectra (NA) per time point<br>ii. Averaging method (eg block-wise or moving average)<br>iii. Total number of spectra (acquired/in time series)	700 transients, alternating edit-ON/-OFF with CHESS suppression pulses disabled in every third transient averages.<br>Of these, 220 were preceded by task stimulus (grouped into 30-second task-ON blocks, separated by 60-second task-OFF blocks)<br>Further subdivided into even time bins (roughly 73 averages each), and according to stimulus and response (varying sizes)
f. Additional sequence parameters (spectral width in Hz, number of spectral points, frequency offsets)<br>If STEAM:, mixing time (T_M)<br>If MRSI: 2D or 3D, FOV in all directions, matrix size, acceleration factors, sampling method	Spectral width 5000Hz, 4096 data points<br>15 ms editing pulses at 1.9 ppm (edit-ON) and 7.46 ppm (edit-OFF)
g. Water suppression method	CHESS
h. Shimming method, reference peak, and thresholds for “acceptance of shim” chosen	Vendor default prescan (double-echo GRE)
i. Triggering or motion correction method (respiratory, peripheral, cardiac triggering, incl. device used and delays)	MRS served as trigger source for the functional paradigm
**3. Data analysis methods and outputs**	
a. Analysis software	Gannet 3.1, with in-house methods to extract functional subsets between GannetLoad and GannetFit modules.
b. Processing steps deviating from quoted reference or product	Spectra extracted from decomposition of full set of transients, described in section 2.2
c. Output measure (eg absolute concentration, institutional units, ratio), processing steps deviating from quoted reference or product	Water-referenced estimates for GABA+ and Glx, with adjustment for voxel tissue content
d. Quantification references and assumptions, fitting model assumptions	N/A
**4. Data quality**	
a. Reported variables (SNR, linewidth (with reference peaks))	SNR NAA: 97.7±11.1/95.7±12.9; 41.9±3.95/40.8±3.79<br>FWHM NAA (Hz): 6.91±0.73/6.97±0.73; 7.22±0.61/7.27±0.76<br>FWHM GABA+ (Hz): 18±1.82/16±2.5; 18.8±1.59/16.8±2.69<br>Denoted median±MAD, patient/control, task-OFF; task-ON
b. Data exclusion criteria	FWHM linewidth > 12 Hz (NAA_{diff}) or > 30 Hz ($GABA+_{diff}$)<br>SNR extraordinarily low, < 20 (NAA_{diff})<br>Extreme outliers (> 5 x median absolute deviation) for $GABA+_{diff}$ or Glx_{diff} estimate;<br>See section 2.2
c. Quality measures of postprocessing model fitting (eg CRLB, goodness of fit, SD of residual)	Strong outlier removal and robust statistics only: individual fits to event-related data expected to be of lower quality than non-functional MRS.
d. Sample spectrum	See Figure 4

Supplementary Table 2 MRSinMRS checklist[19] summarising key details of the MRS acquisition

C Supplementary Results

Session	Group	N subjects	Stimulus Type	N stimuli (per subj.)	Achieved ISI mean (ms)	Achieved ISI SD (ms)	RT (ms)	RA (% correct)	RA/RT
fMRS	Control	51	Congruent	132	1499.6 ± 5.6	107.3 ± 6.8	428.7 ± 38.9 ***/n.s./n.s.	98.5 ± 3.5 ***/n.s./***	.228 ± .022 ***/n.s./***
			Incongruent	88	1504.5 ± 8.7	108.2 ± 5.1	522.2 ± 51.0 ***/n.s./n.s.	84.1 ± 14.8 ***/***/***	.159 ± .029 ***/***/***
			Difference		(incompatibility slowing)		92.7 ± 23.3 ***/n.s./n.s.		
	Patient	51	Congruent	132	1501.7 ± 7.0	107.0 ± 10.4	451.6 ± 77.8 ***/n.s./n.s.	86.4 ± 18.7 ***/n.s./***	.182 ± .044 ***/n.s./***
			Incongruent	88	1500.9 ± 10.7	106.0 ± 12.9	538.0 ± 113.0 ***/n.s./n.s.	54.5 ± 23.8 ***/***/***	.100 ± .041 ***/***/***
			Difference		(incompatibility slowing)		103.8 ± 51.0 ***/n.s./n.s.		
fMRI	Control	51	Congruent	72	1496.9 ± 7.5	102.8 ± 6.1	437.3 ± 39.0 ***/n.s./n.s.	98.6 ± 3.1 ***/n.s./***	.225 ± .021 ***/n.s./***
			Incongruent	48	1503.0 ± 11.0	102.7 ± 7.1	520.5 ± 49.4 ***/n.s./n.s.	89.6 ± 13.7 ***/***/***	.161 ± .025 ***/***/***
			Difference		(incompatibility slowing)		84.7 ± 21.0 ***/n.s./n.s.		
	Patient	49	Congruent	72	1498.5 ± 7.1	101.6 ± 6.2	454.7 ± 72.6 ***/n.s./n.s.	93.1 ± 17.3 ***/n.s./***	.193 ± .039 ***/n.s./***
			Incongruent	48	1501.3 ± 9.3	103.4 ± 8.0	561.0 ± 91.6 ***/n.s./n.s.	64.6 ± 24.1 ***/***/***	.119 ± .038 ***/***/***
			Difference		(incompatibility slowing)		90.7 ± 41.8 ***/n.s./n.s.		

*Supplementary Table 3 Behavioural outcomes from the Flanker task; values are quoted as Median +/- Median Absolute Deviation (MAD) of per-subject outcomes. Significant differences are indicated between stimulus type, session and group (denoted type/session/group, *** $p_{holm}<0.001$, ** $p_{holm}<0.01$, * $p_{holm}<0.05$, n.s. not significant). ISI: Inter-stimulus interval, RA: Response Accuracy, RT: Response Time*

	Control		Patient	
	task-OFF	task-ON	task-OFF	task-ON
SNR NAA	97.7 ± 11.1	41.9 ± 3.95	95.7 ± 12.9	40.8 ± 3.79
FWHM Cho/Cr *before* linewidth matching n.s.	6.84 ± 0.569	6.72 ± 0.619	7.07 ± 0.474	7.02 ± 0.518
FWHM Cho/Cr *after* linewidth matching n.s.	6.88 ± 0.569	7.03 ± 0.651	7.24 ± 0.54	7.23 ± 0.509
FWHM NAA (Hz)	6.91 ± 0.729	6.97 ± 0.729	7.22 ± 0.608	7.27 ± 0.764
FWHM GABA+ (Hz)	18 ± 1.82	16.3 ± 2.5	18.8 ± 1.59	16.8 ± 2.69
FWHM Glx (Hz)	12.1 ± 1.11	12.7 ± 1.47	12.3 ± 0.9	12.5 ± 1.49
GABA+ (i.u., ≈mM)	2.9 ± 0.367	2.88 ± 0.46	2.87 ± 0.372	2.79 ± 0.594
Glx (i.u., ≈mM)	14.7 ± 1.35	15.7 ± 2.03	14.2 ± 1.33	14.9 ± 2.39

Supplementary Table 4 Quality metrics (signal-to-noise ratio, SNR, and linewidth as full width at half-maximum, FWHM) and concentration estimates from the fMRS analysis, task-ON vs task-OFF, presented as median ± MAD.

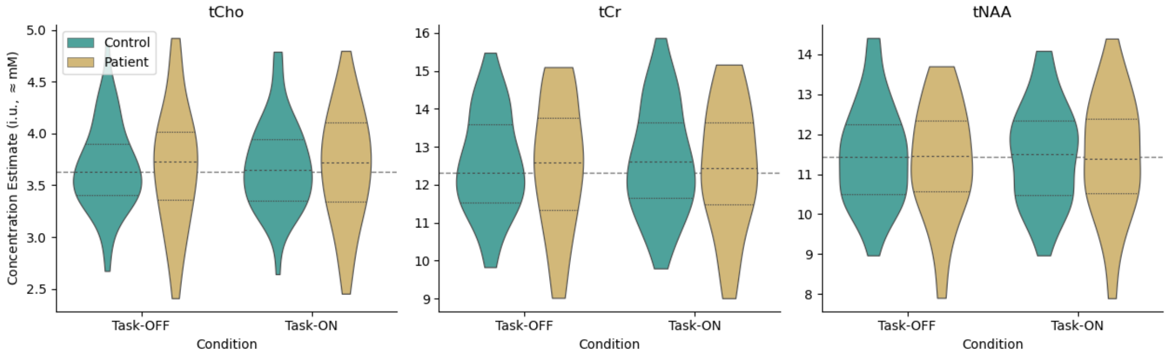

Supplementary Figure 2 Concentration estimates for other metabolites according to group and condition (obtained from the edit-OFF sub-spectrum using the Gannet peak-fitting model)

C.1 Regression Modelling Outcomes: Glx

Associations between baseline Glx estimate, BOLD signal strength and interactions with patient and control groups, with voxel grey matter fraction fGM and age as covariates (ie, `Glx ~ C(group)*BOLD + fGM + age`), after removing outlier observations:

OLS Regression Results

Dep. Variable:	Glx_ConcIU_rest	R-squared:	0.170
Model:	OLS	Adj. R-squared:	0.118
Method:	Least Squares	F-statistic:	3.268
Date:	Thu, 03 Apr 2025	Prob (F-statistic):	0.00978
Time:	17:30:03	Log-Likelihood:	-166.69
No. Observations:	86	AIC:	345.4
Df Residuals:	80	BIC:	360.1
Df Model:	5		
Covariance Type:	nonrobust		

	coef	std err	t	P>|t|	[0.025	0.975]
Intercept	17.2474	3.050	5.656	0.000	11.179	23.316
**C(group, Treatment("control"))[T.patient]**	**-2.2288**	**0.668**	**-3.335**	**0.001**	**-3.559**	**-0.899**
**BOLD**	**-0.3792**	**0.147**	**-2.586**	**0.012**	**-0.671**	**-0.087**
**C(group, Treatment("control"))[T.patient]:BOLD**	**0.5402**	**0.239**	**2.256**	**0.027**	**0.064**	**1.017**
Age	-0.0307	0.024	-1.306	0.195	-0.078	0.016
fGM	-0.5869	4.264	-0.138	0.891	-9.073	7.899

Omnibus:	1.501	Durbin-Watson:	2.182
Prob(Omnibus):	0.472	Jarque-Bera (JB):	1.541
Skew:	0.264	Prob(JB):	0.463
Kurtosis:	2.611	Cond. No.	907.

Glx estimate in relation to group (patient, control), task status (task-OFF, task-ON), with grey matter fraction and age as covariates and subject as the grouping variable (ie, `Glx ~ C(group)*C(task_state) + fGM + age`), after filtering observations with strong residuals:

Model:	MixedLM	Dependent Variable:	value
No. Observations:	197	Method:	REML
No. Groups:	99	Scale:	4.0263
Min. group size:	1	Log-Likelihood:	-467.2020
Max. group size:	2	Converged:	Yes
Mean group size:	2.0		

	Coef.	Std.Err.	z	P>|z|	[0.025	0.975]
Intercept	14.994	3.876	3.868	0.000	7.396	22.591
**C(task_state)[T.True]**	**1.088**	**0.409**	**2.662**	**0.008**	**0.287**	**1.888**
C(group, Treatment("control"))[T.patient]	-0.947	0.568	-1.666	0.096	-2.061	0.167
C(task_state)[T.True]:C(group, Treatment("control"))[T.pat...	-0.351	0.573	-0.614	0.540	-1.474	0.771
fGM	0.320	5.466	0.059	0.953	-10.393	11.033
Age	-0.004	0.031	-0.127	0.899	-0.064	0.056
subject Var	3.901	0.602				

C.2 Regression Modelling Outcomes: GABA+

Associations between baseline GABA+ estimate, BOLD signal strength and interactions with patient and control groups, with voxel grey matter fraction fGM and age as covariates (ie, `GABA ~ C(group)*BOLD + fGM + age`), after removing outlier observations:

OLS Regression Results

Dep. Variable:	GABA_rest	R-squared:	0.063
Model:	OLS	Adj. R-squared:	0.003
Method:	Least Squares	F-statistic:	1.044
Date:	Thu, 03 Apr 2025	Prob (F-statistic):	0.398
Time:	17:29:29	Log-Likelihood:	-2.0925
No. Observations:	83	AIC:	16.19
Df Residuals:	77	BIC:	30.70
Df Model:	5		
Covariance Type:	nonrobust		

	coef	std err	t	P>|t|	[0.025	0.975]
Intercept	1.7780	0.549	3.239	0.002	0.685	2.871
C(group, Treatment("control"))[T.patient]	-0.1393	0.107	-1.300	0.198	-0.353	0.074
**BOLD**	**-0.0489**	**0.024**	**-2.077**	**0.041**	**-0.096**	**-0.002**
C(group, Treatment("control"))[T.patient]:BOLD	0.0499	0.038	1.316	0.192	-0.026	0.125
Age	-0.0017	0.004	-0.411	0.682	-0.010	0.007
fGM	0.0793	0.765	0.104	0.918	-1.444	1.602

Omnibus:	1.731	Durbin-Watson:	2.333
Prob(Omnibus):	0.421	Jarque-Bera (JB):	1.764
Skew:	0.313	Prob(JB):	0.414
Kurtosis:	2.657	Cond. No.	1.05e+03

GABA+ estimate in relation to group (patient, control), task status (task-OFF, task-ON), with grey matter fraction and age as covariates and subject as the grouping variable (ie, `GABA ~ C(group)*C(task_state) + fGM + age`), after filtering observations with strong residuals:

Mixed Linear Model Regression Results

Model:	MixedLM	Dependent Variable:	value
No. Observations:	197	Method:	REML
No. Groups:	99	Scale:	0.0799
Min. group size:	1	Log-Likelihood:	-86.0570
Max. group size:	2	Converged:	Yes
Mean group size:	2.0		

	Coef.	Std.Err.	z	P>|z|	[0.025	0.975]
Intercept	1.575	0.507	3.107	0.002	0.581	2.569
C(task_state)[T.True]	-0.010	0.057	-0.179	0.858	-0.122	0.102
C(group, Treatment("control"))[T.patient]	-0.038	0.076	-0.497	0.619	-0.186	0.111
C(task_state)[T.True]:C(group, Treatment("control"))[T.pat..	-0.083	0.081	-1.024	0.306	-0.241	0.076
fGM	0.218	0.715	0.305	0.760	-1.183	1.620
Age	-0.002	0.004	-0.413	0.679	-0.010	0.006
subject Var	0.062	0.074				

C.3 Regression Modelling Outcomes: Glx, exploratory, SZ subgroup

SZ subgroup analysis: associations between baseline Glx estimate, BOLD signal strength and interactions with patient and control groups, with voxel grey matter fraction fGM and age as covariates (ie, `Glx ~ C(group)*BOLD + fGM + age`), after removing outlier observations:

```
                            OLS Regression Results                            
==============================================================================
Dep. Variable:        Glx_ConcIU_rest   R-squared:                       0.133
Model:                            OLS   Adj. R-squared:                  0.053
Method:                 Least Squares   F-statistic:                     1.657
Date:                Thu, 03 Apr 2025   Prob (F-statistic):              0.161
Time:                        17:30:15   Log-Likelihood:                -125.18
No. Observations:                  60   AIC:                             262.4
Df Residuals:                      54   BIC:                             274.9
Df Model:                           5                                         
Covariance Type:            nonrobust                                         
====================================================================================================
                                                     coef    std err          t      P>|t|      [0.025      0.975]
----------------------------------------------------------------------------------------------------
Intercept                                         14.9722      4.759      3.146      0.003       5.430      24.514
C(group, Treatment("control"))[T.patient]         -1.8158      0.993     -1.828      0.073      -3.807       0.175
BOLD                                               0.0027      0.226      0.012      0.991      -0.451       0.456
C(group, Treatment("control"))[T.patient]:BOLD     0.1701      0.355      0.479      0.634      -0.542       0.883
Age                                               -0.0026      0.039     -0.067      0.947      -0.081       0.075
fGM                                                0.7334      6.474      0.113      0.910     -12.246      13.713
==============================================================================
Omnibus:                        6.564   Durbin-Watson:                   2.568
Prob(Omnibus):                  0.038   Jarque-Bera (JB):                5.864
Skew:                           0.585   Prob(JB):                       0.0533
Kurtosis:                       3.987   Cond. No.                         988.
==============================================================================
```

SZ subgroup analysis: Glx estimate in relation to group (patient, control), task status (task-OFF, task-ON), with grey matter fraction and age as covariates and subject as the grouping variable (ie, `Glx ~ C(group)*C(task_state) + fGM + age`), after filtering observations with strong residuals:

```
                         Mixed Linear Model Regression Results
======================================================================================
Model:                      MixedLM            Dependent Variable:           value    
No. Observations:           137                Method:                       REML     
No. Groups:                 69                 Scale:                        3.9017   
Min. group size:            1                  Log-Likelihood:               -324.6633
Max. group size:            2                  Converged:                    Yes      
Mean group size:            2.0                                                       
--------------------------------------------------------------------------------------
                                                       Coef.  Std.Err.   z    P>|z| [0.025 0.975]
--------------------------------------------------------------------------------------
Intercept                                              10.090    5.547  1.819 0.069 -0.781 20.961
C(task_state)[T.True]                                   0.667    0.485  1.377 0.168 -0.282  1.617
C(group, Treatment("control"))[T.patient]              -1.161    0.704 -1.649 0.099 -2.540  0.219
C(task_state)[T.True]:C(group, Treatment("control"))[T.pat..  0.310    0.677  0.458 0.647 -1.017  1.636
fGM                                                     5.905    7.601  0.777 0.437 -8.992 20.803
Age                                                     0.050    0.045  1.130 0.258 -0.037  0.138
subject Var                                             4.512    0.807                            
======================================================================================
```

C.4 Regression Modelling Outcomes: GABA+, exploratory, SZ subgroup

SZ subgroup analysis: associations between baseline GABA+ estimate, BOLD signal strength and interactions with patient and control groups, with voxel grey matter fraction fGM and age as covariates (ie, `GABA ~ C(group)*BOLD + fGM + age`), after removing outlier observations:

OLS Regression Results

Dep. Variable:	GABA_rest	R-squared:	0.188
Model:	OLS	Adj. R-squared:	0.109
Method:	Least Squares	F-statistic:	2.401
Date:	Thu, 03 Apr 2025	Prob (F-statistic):	0.0493
Time:	17:29:41	Log-Likelihood:	5.3846
No. Observations:	58	AIC:	1.231
Df Residuals:	52	BIC:	13.59
Df Model:	5		
Covariance Type:	nonrobust		

	coef	std err	t	P>|t|	[0.025	0.975]
Intercept	1.1985	0.541	2.217	0.031	0.114	2.283
C(group, Treatment("control"))[T.patient]	-0.1595	0.110	-1.445	0.154	-0.381	0.062
**BOLD**	**-0.0624**	**0.027**	**-2.338**	**0.023**	**-0.116**	**-0.009**
C(group, Treatment("control"))[T.patient]:BOLD	0.0504	0.041	1.218	0.229	-0.033	0.134
Age	-0.0021	0.004	-0.469	0.641	-0.011	0.007
fGM	1.1104	0.753	1.474	0.146	-0.401	2.622

Omnibus:	1.199	Durbin-Watson:	2.039
Prob(Omnibus):	0.549	Jarque-Bera (JB):	1.058
Skew:	0.130	Prob(JB):	0.589
Kurtosis:	2.392	Cond. No.	970.

SZ subgroup analysis: GABA+ estimate in relation to group (patient, control), task status (task-OFF, task-ON), with grey matter fraction and age as covariates and subject as the grouping variable (ie, `GABA ~ C(group)*C(task_state) + fGM + age`), after filtering observations with strong residuals:

Mixed Linear Model Regression Results

Model:	MixedLM	Dependent Variable:	value
No. Observations:	137	Method:	REML
No. Groups:	69	Scale:	0.0620
Min. group size:	1	Log-Likelihood:	-52.8717
Max. group size:	2	Converged:	Yes
Mean group size:	2.0		

	Coef.	Std.Err.	z	P>|z|	[0.025	0.975]
Intercept	1.005	0.691	1.455	0.146	-0.349	2.359
C(task_state)[T.True]	-0.089	0.060	-1.479	0.139	-0.208	0.029
C(group, Treatment("control"))[T.patient]	-0.011	0.088	-0.126	0.900	-0.183	0.161
C(task_state)[T.True]:C(group, Treatment("control"))[T.pat..	0.038	0.085	0.451	0.652	-0.129	0.206
fGM	0.985	0.947	1.039	0.299	-0.872	2.842
Age	0.001	0.006	0.220	0.826	-0.010	0.012
subject Var	0.070	0.100				

C.5 Regression Modelling Outcomes: Glx, exploratory, OD subgroup

OD subgroup analysis: associations between baseline Glx estimate, BOLD signal strength and interactions with patient and control groups, with voxel grey matter fraction fGM and age as covariates (ie, `Glx ~ C(group)*BOLD + fGM + age`), after removing outlier observations:

```
                            OLS Regression Results
==============================================================================
Dep. Variable:        Glx_ConcIU_rest   R-squared:                       0.596
Model:                            OLS   Adj. R-squared:                  0.489
Method:                 Least Squares   F-statistic:                     5.597
Date:                Thu, 03 Apr 2025   Prob (F-statistic):            0.00246
Time:                        17:30:26   Log-Likelihood:                -35.090
No. Observations:                  25   AIC:                             82.18
Df Residuals:                      19   BIC:                             89.49
Df Model:                           5
Covariance Type:            nonrobust
======================================================================================================
                                                    coef  std err        t    P>|t|   [0.025    0.975]
------------------------------------------------------------------------------------------------------
Intercept                                        25.0736    3.267    7.674    0.000   18.235    31.912
C(group, Treatment("control"))[T.patient]        -2.6428    0.784   -3.370    0.003   -4.284    -1.002
BOLD                                             -0.5544    0.147   -3.763    0.001   -0.863    -0.246
C(group, Treatment("control"))[T.patient]:BOLD    1.0832    0.278    3.901    0.001    0.502     1.664
Age                                              -0.0323    0.023   -1.395    0.179   -0.081     0.016
fGM                                             -13.2218    4.960   -2.665    0.015  -23.604    -2.840
==============================================================================
Omnibus:                        0.385   Durbin-Watson:                   1.959
Prob(Omnibus):                  0.825   Jarque-Bera (JB):                0.460
Skew:                          -0.253   Prob(JB):                        0.794
Kurtosis:                       2.570   Cond. No.                         869.
==============================================================================
```

OD subgroup analysis: Glx estimate in relation to group (patient, control), task status (task-OFF, task-ON), with grey matter fraction and age as covariates and subject as the grouping variable (ie, `Glx ~ C(group)*C(task_state) + fGM + age`), after filtering observations with strong residuals:

```
                         Mixed Linear Model Regression Results
=========================================================================================================
Model:                              MixedLM              Dependent Variable:                 value
No. Observations:                   60                   Method:                             REML
No. Groups:                         30                   Scale:                              4.1461
Min. group size:                    2                    Log-Likelihood:                     -134.4549
Max. group size:                    2                    Converged:                          Yes
Mean group size:                    2.0
---------------------------------------------------------------------------------------------------------
                                                             Coef.  Std.Err.    z    P>|z|  [0.025 0.975]
---------------------------------------------------------------------------------------------------------
Intercept                                                    19.241    5.183  3.712 0.000   9.082 29.401
C(task_state)[T.True]                                         2.028    0.744  2.728 0.006   0.571  3.485
C(group, Treatment("control"))[T.patient]                    -0.255    0.960 -0.265 0.791  -2.135  1.626
C(task_state)[T.True]:C(group, Treatment("control"))[T.pat. -1.854    1.051 -1.763 0.078  -3.915  0.207
fGM                                                          -4.485    7.645 -0.587 0.557 -19.470 10.500
Age                                                          -0.064    0.040 -1.588 0.112  -0.142  0.015
subject Var                                                   2.586    0.881
=========================================================================================================
```

C.6 Regression Modelling Outcomes: GABA+, exploratory, OD subgroup

OD subgroup analysis: associations between baseline GABA+ estimate, BOLD signal strength and interactions with patient and control groups, with voxel grey matter fraction fGM and age as covariates (ie, `GABA ~ C(group)*BOLD + fGM + age`), after removing outlier observations:

```
                            OLS Regression Results
==============================================================================
Dep. Variable:              GABA_rest   R-squared:                       0.375
Model:                            OLS   Adj. R-squared:                  0.211
Method:                 Least Squares   F-statistic:                     2.280
Date:                Thu, 03 Apr 2025   Prob (F-statistic):             0.0878
Time:                        17:29:52   Log-Likelihood:                 1.1694
No. Observations:                  25   AIC:                             9.661
Df Residuals:                      19   BIC:                             16.97
Df Model:                           5
Covariance Type:            nonrobust
===================================================================================================
                                                    coef    std err          t      P>|t|      [0.025      0.975]
---------------------------------------------------------------------------------------------------
Intercept                                         3.4930      0.841      4.154      0.001       1.733       5.253
C(group, Treatment("control"))[T.patient]        -0.1459      0.229     -0.637      0.532      -0.625       0.334
BOLD                                             -0.0257      0.050     -0.516      0.612      -0.130       0.079
C(group, Treatment("control"))[T.patient]:BOLD    0.1116      0.068      1.632      0.119      -0.032       0.255
Age                                              -0.0093      0.007     -1.314      0.204      -0.024       0.005
fGM                                              -2.5121      1.107     -2.269      0.035      -4.829      -0.195
==============================================================================
Omnibus:                        8.365   Durbin-Watson:                   2.096
Prob(Omnibus):                  0.015   Jarque-Bera (JB):                6.545
Skew:                           0.925   Prob(JB):                       0.0379
Kurtosis:                       4.691   Cond. No.                         822.
==============================================================================
```

OD subgroup analysis: GABA+ estimate in relation to group (patient, control), task status (task-OFF, task-ON), with grey matter fraction and age as covariates and subject as the grouping variable (ie, `GABA ~ C(group)*C(task_state) + fGM + age`), after filtering observations with strong residuals:

```
                       Mixed Linear Model Regression Results
=====================================================================================================
Model:                         MixedLM              Dependent Variable:                  value
No. Observations:              60                   Method:                              REML
No. Groups:                    30                   Scale:                               0.1123
Min. group size:               2                    Log-Likelihood:                      -33.7669
Max. group size:               2                    Converged:                           Yes
Mean group size:               2.0
----------------------------------------------------------------------------------------------------
                                                            Coef.  Std.Err.   z    P>|z| [0.025 0.975]
----------------------------------------------------------------------------------------------------
Intercept                                                    2.021    0.753  2.682 0.007  0.544  3.497
C(task_state)[T.True]                                        0.169    0.122  1.381 0.167 -0.071  0.409
C(group, Treatment("control"))[T.patient]                   -0.078    0.145 -0.541 0.589 -0.363  0.206
C(task_state)[T.True]:C(group, Treatment("control"))[T.pat  -0.357    0.173 -2.063 0.039 -0.696 -0.018
fGM                                                         -0.418    1.110 -0.376 0.707 -2.594  1.758
Age                                                         -0.003    0.006 -0.491 0.623 -0.014  0.009
subject Var                                                  0.042    0.113
====================================================================================================
```

C.7 Regression Modelling Outcomes: exploratory, antipsychotic effects

Exploratory analysis investigating associations with prescribed DDD of antipsychotic medication (ATC[18] N05A) in the SZ subgroup: `Glx ~ C(task_state) * DDD_Antipsychotics + fGM + Age + PANSS_total_positive`, after filtering observations with strong residuals.

Glx

Mixed Linear Model Regression Results

Model:	MixedLM	Dependent Variable:	value
No. Observations:	52	Method:	REML
No. Groups:	26	Scale:	3.5629
Min. group size:	2	Log-Likelihood:	-114.7527
Max. group size:	2	Converged:	Yes
Mean group size:	2.0		

	Coef.	Std.Err.	z	P>|z|	[0.025	0.975]
Intercept	7.687	9.725	0.790	0.429	-11.374	26.748
C(task_state)[T.True]	0.951	0.847	1.123	0.261	-0.709	2.610
DDD_Antipsychotics	0.364	0.456	0.798	0.425	-0.530	1.258
C(task_state)[T.True]:DDD_Antipsychotics	0.151	0.484	0.311	0.756	-0.798	1.099
fGM	7.900	13.255	0.596	0.551	-18.079	33.879
Age	0.018	0.065	0.282	0.778	-0.109	0.145
PANSS_total_positive	0.003	0.075	0.041	0.967	-0.145	0.151
subject Var	2.565	0.973				

GABA+

Mixed Linear Model Regression Results

Model:	MixedLM	Dependent Variable:	value
No. Observations:	51	Method:	REML
No. Groups:	26	Scale:	0.0560
Min. group size:	1	Log-Likelihood:	-22.6641
Max. group size:	2	Converged:	Yes
Mean group size:	2.0		

	Coef.	Std.Err.	z	P>|z|	[0.025	0.975]
Intercept	1.259	1.318	0.955	0.340	-1.325	3.842
C(task_state)[T.True]	-0.153	0.108	-1.415	0.157	-0.366	0.059
**DDD_Antipsychotics**	**0.147**	**0.060**	**2.433**	**0.015**	**0.029**	**0.265**
C(task_state)[T.True]:DDD_Antipsychotics	0.035	0.061	0.569	0.570	-0.085	0.155
fGM	0.198	1.793	0.110	0.912	-3.317	3.713
Age	-0.001	0.009	-0.058	0.954	-0.018	0.017
PANSS_total_positive	0.003	0.010	0.297	0.767	-0.017	0.023
subject Var	0.051	0.142				

C.8 Regression Modelling Outcomes: exploratory, stimulus/response binning

Exploratory analyses performed to investigate any difference in metabolite response to specific task conditions (congruent vs incongruent stimuli, the latter separated into correct and incorrect responses): `Glx ~ C(group)*C(task_stimulus_and_accuracy) + fGM + age`

Glx

Mixed Linear Model Regression Results

Model:	MixedLM	Dependent Variable:	value
No. Observations:	234	Method:	REML
No. Groups:	77	Scale:	4.8686
Min. group size:	1	Log-Likelihood:	-529.3450
Max. group size:	4	Converged:	Yes
Mean group size:	3.0		

	Coef.	Std.Err.	z	P>|z|	[0.025	0.975]
Intercept	14.321	2.842	5.039	0.000	8.750	19.891
C(condition)[T.Glx_cong]	0.084	0.548	0.153	0.878	-0.990	1.158
C(condition)[T.Glx_incong_acc]	0.965	0.612	1.577	0.115	-0.234	2.164
C(condition)[T.Glx_incong_inacc]	0.359	0.640	0.560	0.575	-0.896	1.613
C(group, Treatment("control"))[T.patient]	-0.137	0.562	-0.244	0.807	-1.239	0.964
C(condition)[T.Glx_cong]:C(group)[T.patient]	0.356	0.738	0.483	0.629	-1.091	1.803
C(condition)[T.Glx_incong_acc]:C(group)[T.patient]	-0.436	0.829	-0.526	0.599	-2.061	1.190
C(condition)[T.Glx_incong_inacc]:C(group)[T.patient]	-1.458	0.897	-1.626	0.104	-3.216	0.299
fGM	0.335	3.948	0.085	0.932	-7.403	8.074
Age	-0.008	0.023	-0.345	0.730	-0.054	0.038
subject Var	0.922	0.230				

GABA+

Mixed Linear Model Regression Results

Model:	MixedLM	Dependent Variable:	value
No. Observations:	266	Method:	REML
No. Groups:	77	Scale:	0.1409
Min. group size:	1	Log-Likelihood:	-153.6877
Max. group size:	4	Converged:	Yes
Mean group size:	3.5		

	Coef.	Std.Err.	z	P>|z|	[0.025	0.975]
Intercept	1.894	0.487	3.892	0.000	0.940	2.848
C(condition)[T.GABA_cong]	-0.023	0.092	-0.252	0.801	-0.204	0.158
C(condition)[T.GABA_incong_acc]	-0.116	0.098	-1.181	0.237	-0.308	0.076
C(condition)[T.GABA_incong_inacc]	-0.193	0.101	-1.914	0.056	-0.390	0.005
C(group, Treatment("control"))[T.patient]	-0.002	0.097	-0.016	0.987	-0.191	0.188
C(condition)[T.GABA_cong]:C(group)[T.patient]	0.046	0.123	0.375	0.708	-0.194	0.286
C(condition)[T.GABA_incong_acc]:C(group)[T.patient]	0.019	0.131	0.143	0.886	-0.239	0.276
C(condition)[T.GABA_incong_inacc]:C(group)[T.patient]	0.084	0.137	0.613	0.540	-0.184	0.351
fGM	-0.283	0.675	-0.420	0.675	-1.606	1.040
Age	-0.003	0.004	-0.771	0.441	-0.011	0.005
subject Var	0.035	0.040				

C.9 Regression Modelling Outcomes: exploratory, stimulus to response time

Exploratory analyses performed to investigate temporal characteristics of the metabolite response (between spectra binned by $T_{S\text{-}A}$): `Glx ~ C(group)*C(time_stimulus_to_acquisition) + fGM + age`.

Glx

Model:	MixedLM	Dependent Variable:	Glx
No. Observations:	339	Method:	REML
No. Groups:	99	Scale:	6.7223
Min. group size:	1	Log-Likelihood:	-856.5552
Max. group size:	4	Converged:	Yes
Mean group size:	3.4		

	Coef.	Std.Err.	z	P>|z|	[0.025	0.975]
Intercept	16.591	4.098	4.048	0.000	8.558	24.623
**C(time_pt)[T.100]**	**1.622**	**0.563**	**2.880**	**0.004**	**0.518**	**2.726**
C(time_pt)[T.183]	0.948	0.556	1.705	0.088	-0.142	2.039
C(time_pt)[T.267]	0.178	0.544	0.328	0.743	-0.888	1.244
C(group)[T.patient]	-0.927	0.678	-1.367	0.172	-2.257	0.402
C(time_pt)[T.100]:C(group)[T.patient]	-1.296	0.803	-1.613	0.107	-2.871	0.279
C(time_pt)[T.183]:C(group)[T.patient]	0.211	0.798	0.265	0.791	-1.354	1.776
C(time_pt)[T.267]:C(group)[T.patient]	0.236	0.772	0.306	0.760	-1.277	1.750
fGM	-3.023	5.765	-0.524	0.600	-14.322	8.275
Age	0.009	0.033	0.279	0.780	-0.055	0.073
subject Var	4.561	0.439				

GABA+

Model:	MixedLM	Dependent Variable:	GABA+
No. Observations:	348	Method:	REML
No. Groups:	99	Scale:	0.1574
Min. group size:	1	Log-Likelihood:	-234.4985
Max. group size:	4	Converged:	Yes
Mean group size:	3.5		

	Coef.	Std.Err.	z	P>|z|	[0.025	0.975]
Intercept	1.246	0.558	2.234	0.025	0.153	2.338
C(time_pt)[T.100]	0.003	0.084	0.033	0.974	-0.162	0.168
C(time_pt)[T.183]	-0.072	0.086	-0.831	0.406	-0.241	0.097
C(time_pt)[T.267]	-0.091	0.083	-1.094	0.274	-0.253	0.072
C(group)[T.patient]	-0.040	0.097	-0.406	0.685	-0.231	0.151
C(time_pt)[T.100]:C(group)[T.patient]	-0.173	0.119	-1.456	0.146	-0.406	0.060
C(time_pt)[T.183]:C(group)[T.patient]	0.022	0.121	0.184	0.854	-0.214	0.259
C(time_pt)[T.267]:C(group)[T.patient]	0.037	0.118	0.309	0.757	-0.195	0.269
fGM	0.735	0.784	0.938	0.348	-0.800	2.271
Age	-0.001	0.004	-0.245	0.806	-0.010	0.008
subject Var	0.078	0.053				

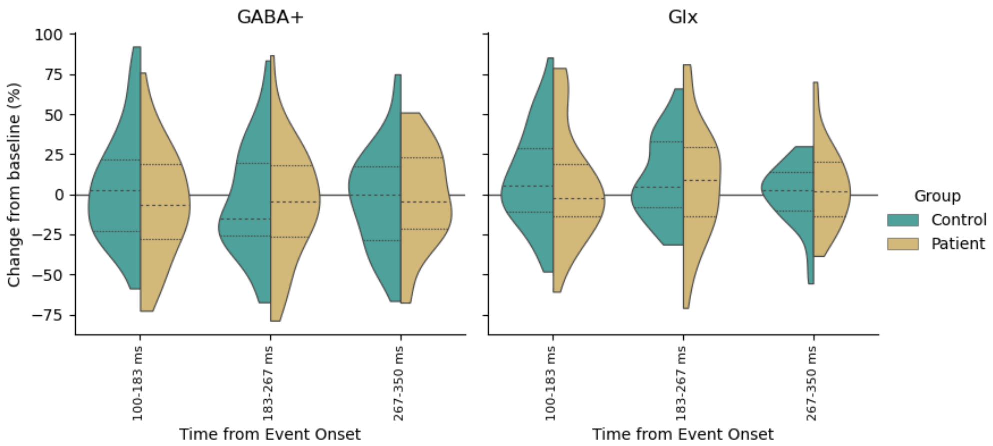

Supplementary Figure 3 Relative change in metabolite concentration estimate, from transients binned according to time from stimulus onset to acquisition

C.10 Exploratory correlational tests

Variables →	PANSS P3			PANSS total positive			PANSS total negative		
↓	r	$p_{unc.}$	p_{holm}	r	$p_{unc.}$	p_{holm}	r	$p_{unc.}$	p_{holm}
Baseline Glx	-0.00 [-0.29, 0.28]	0.974	1	0.01 [-0.28, 0.29]	0.963	1	-0.06 [-0.34, 0.22]	0.661	1
ΔGlx	-0.01 [-0.29, 0.27]	0.945	1	**-0.34 [-0.57,-0.05]**	**0.0228**	**0.457**	-0.01 [-0.29, 0.27]	0.96	1
Baseline GABA+	-0.17 [-0.45, 0.15]	0.293	1	-0.14 [-0.41, 0.15]	0.337	1	0.05 [-0.24, 0.32]	0.744	1
ΔGABA+	-0.15 [-0.41, 0.14]	0.312	1	-0.03 [-0.32, 0.27]	0.858	1	0.07 [-0.22, 0.35]	0.618	1
BOLD-fMRI	-0.08 [-0.38, 0.23]	0.598	1	-0.10 [-0.38, 0.20]	0.508	1	-0.11 [-0.39, 0.18]	0.458	1
Task performance: RA/RT	-0.19 [-0.47, 0.13]	0.241	1	-0.15 [-0.42, 0.14]	0.301	1	-0.28 [-0.52, 0.00]	0.0536	1
Task performance: RT_slowing	-0.24 [-0.49, 0.05]	0.0982	1	-0.24 [-0.49, 0.05]	0.0988	1	**-0.35 [-0.58,-0.07]**	**0.0143**	**0.301**

Supplementary Table 5 Exploratory correlational testing; skipped Spearman correlation with 95% confidence interval

C.11 Supplementary Figures and Tables

Supplementary Figure 1 Medication and mean of the (maximum) prescribed dosage, expressed relative to the defined daily dose (DDD). Size of the points is proportional to the number of patients (N), with darker shading indicating higher dosage (xDDD). 2

Supplementary Figure 2 Concentration estimates for other metabolites according to group and condition (obtained from the edit-OFF sub-spectrum using the Gannet peak-fitting model) 5

Supplementary Figure 3 Relative change in metabolite concentration estimate, from transients binned according to time from stimulus onset to acquisition........ 14

Supplementary Table 1 Diagnoses of patients in the present study, according to ICD-10 criteria 1

Supplementary Table 2 MRSinMRS checklist[19] summarising key details of the MRS acquisition 3

Supplementary Table 3 Behavioural outcomes from the Flanker task; values are quoted as Median +/- Median Absolute Deviation (MAD) of per-subject outcomes. Significant differences are indicated between stimulus type, session and group (denoted type/session/group, *** p_{holm}<0.001, ** p_{holm}<0.01, * p_{holm}<0.05, n.s. not significant). ISI: Inter-stimulus interval, RA: Response Accuracy, RT: Response Time........ 4

Supplementary Table 4 Quality metrics (signal-to-noise ratio, SNR, and linewidth as full width at half-maximum, FWHM) and concentration estimates from the fMRS analysis, task-ON vs task-OFF, presented as median ± MAD........ 5

Supplementary Table 5 Exploratory correlational testing; skipped Spearman correlation with 95% confidence interval 15
